# Diagnosis and Management of Chronic Endometritis: Effects on Postoperative Pregnancy Outcomes in Endometriosis Patients with Proximal Tubal Obstruction Undergoing Hysteroscopic Tubal Catheterization and Laparoscopy

**DOI:** 10.3390/jcm15176845

**Published:** 2026-09-03

**Authors:** Peng Hong, Yixian Han, Wei Huang, Yunwei Ouyang, Huili Zhu, Xinyu Qiao, Ying Long, Jing Tan, Jing Fu

**Affiliations:** 1 Department of Obstetrics and Gynecology, West China Second University Hospital of Sichuan University, Chengdu 610041, China; hongp120@163.com (P.H.); hanyixian4799@163.com (Y.H.); weihuang64@163.com (W.H.); ywoycc@126.com (Y.O.); zhuhuili@scu.edu.cn (H.Z.); qiaoxy20@163.com (X.Q.); longy90@163.com (Y.L.); 15196614193@163.com (J.T.); 2 Key Laboratory of Birth Defects and Related Diseases of Women and Children (Sichuan University), Ministry of Education, West China Second University Hospital of Sichuan University, Chengdu 610041, China; 3 Qamdo People’s Hospital of Tibet Autonomous Region, Qamdo 854000, China

**Keywords:** chronic endometritis, endometriosis, hysteroscopic fallopian tube catheterization, laparoscopy, proximal tubal obstruction, pregnancy outcome

## Abstract

**Background**: This study aims to investigate the impact of chronic endometritis (CE) diagnosis and treatment on pregnancy outcomes during hysteroscopic fallopian tube catheterization combined with laparoscopy in infertility patients with pelvic endometriosis (EMS) and proximal tubal obstruction (PTO). **Methods**: A retrospective study was conducted between July 2020 and March 2022, including patients with PTO who underwent hysteroscopy–laparoscopy. CE was diagnosed via CD138 immunohistochemical staining and treated with oral doxycycline. The study further assessed the prevalence of CE and evaluated the effect of its diagnosis and treatment on pregnancy outcomes. **Results**: A total of 454 participants with PTO were enrolled, of whom 158 had concomitant pelvic EMS. Among EMS patients, 78 underwent CD138 staining for CE diagnosis (Group A), while the rest were classified as Group B. The incidence of CE in Group A was 62.8%. These affected individuals were treated with doxycycline. The natural pregnancy rate was significantly higher in Group A than Group B (46.2% vs. 26.3%, *p* = 0.009). The clinical pregnancy rate and live birth rate were higher in Group A, although the differences were not statistically significant. Additionally, Group A demonstrated a significantly higher cumulative natural pregnancy rate (*p* < 0.05). Notably, the diagnosis and treatment of CE emerged as a protective factor for cumulative natural pregnancy (HR, 1.78; 95% CI, 1.06–2.97; *p* = 0.028). **Conclusions**: In infertility patients with pelvic EMS complicated by PTO undergoing hysteroscopic tube catheterization combined with laparoscopy, prioritizing the diagnosis and treatment of CE is crucial for enhancing natural pregnancy outcomes.

## 1. Introduction

Chronic endometritis (CE) is a persistent inflammatory disorder of the endometrium, characterized by marked plasma cell infiltration within the endometrial stroma [1]. Accumulating evidence has associated CE with infertility and a range of adverse pregnancy outcomes [2], potentially attributed to the chronic inflammatory microenvironment compromising endometrial receptivity [3]. Nonetheless, CE often remains asymptomatic or manifests with nonspecific clinical features, contributing to its frequent underdiagnosis in clinical practice [4]. Currently, the diagnosis of CE relies on histopathological evaluation of endometrial specimens procured via hysteroscopy, with CD138 immunohistochemical staining employed to enhance diagnostic sensitivity and specificity [5].

Endometriosis (EMS), defined as the ectopic presence of endometrial glands and stroma outside the uterine cavity, afflicts approximately 20–50% of infertile women [6]. Through multifactorial mechanisms involving immune dysregulation, cytokine activity, and hormonal imbalance, EMS can disrupt tubal anatomy and function, ultimately leading to proximal tubal obstruction and infertility [7]. Hysteroscopic fallopian tube catheterization combined with laparoscopy represents an effective therapeutic approach for proximal tubal obstruction, as it restores tubal patency and promotes postoperative natural conception [8].

Both pelvic EMS and CE are characterized by chronic inflammatory responses involving altered cytokine signaling, immune-cell trafficking, and potential dysbiosis of the reproductive tract microbiota [9,10]. Accordingly, previous studies have investigated their potential association. Women with EMS have been reported to exhibit a higher prevalence of aberrant uterine microbiota [11] and CE [12,13,14]. Moreover, patients with CE are more likely to present with concomitant EMS and tubal obstruction [15]. Furthermore, some evidence suggests that treatment of CE may enhance pregnancy outcomes in women with endometriosis-related infertility [14]. However, the available evidence remains inconsistent. Clinically, if EMS is associated with an increased risk of CE, targeted preoperative CD138 screening and treatment could potentially improve postoperative pregnancy outcomes; conversely, if CE is not clinically relevant in this population, routine screening may impose additional costs and antibiotic exposure without reproductive benefit.

Nevertheless, limited evidence is available regarding the prevalence and clinical significance of CE among infertile women with coexisting EMS and proximal tubal obstruction. Whether preoperative screening and treatment of CE is associated with improve spontaneous pregnancy rates following combined hysteroscopic and laparoscopic tubal recanalization remains unclear. Therefore, this study aimed to investigate the prevalence of CE and its association with postoperative pregnancy outcomes in this population.

## 2. Materials and Methods

### 2.1. Study Population

A retrospective analysis was conducted on 454 patients who underwent hysteroscopic fallopian tube catheterization combined with laparoscopy for proximal tubal obstruction-related infertility in the West China Second Hospital between July 2020 and March 2022. The study was approved by the Ethics Committee of West China Second Hospital (Approval No. 2024077). The inclusion criteria were as follows: (1) Age between 18 and 35 years; (2) experiencing unprotected sexual intercourse without conception for over 12 months; (3) regular menstrual cycles; (4) documented regular ovulation; (5) normal ovarian reserve, defined as anti-Müllerian hormone (AMH) > 1.1 ng/mL; (6) proximal tubal obstruction confirmed by hysterosalpingography. Exclusion criteria included: (1) Other identifiable causes of infertility, such as male factors; (2) benign uterine pathologies, such as intrauterine adhesions or submucosal fibroids; (3) congenital uterine anomalies; (4) endocrine disorders affecting ovulation, such as premature ovarian insufficiency or polycystic ovary syndrome.

### 2.2. Study Procedures

Following clinical assessment of surgical indications, all patients underwent surgery 3–7 days after the onset of menstruation. Mild-to-moderate tubal damage was addressed intraoperatively through pelvic adhesiolysis or tubal repair/reconstruction. Biopsy and electrocoagulation were performed for pelvic EMS lesions. Cystectomy with subsequent pathological examination was carried out for ovarian endometriosis (OMA). The stage of EMS was determined using the revised American Fertility Society (r-AFS) classification system [16]. Endometriotic lesions were categorized into three phenotypes: superficial peritoneal EMS (SUP), OMA, and deeply infiltrating EMS (DIE) [17]. As these phenotypes often coexist, patients were assigned to the group corresponding to the most severe lesion, ranked in order of increasing severity as SUP, OMA, and DIE [18]. Additionally, intraoperative hydrotubation was performed to confirm proximal tubal obstruction. If no methylene blue spillage was observed from one fallopian tube or if bilateral spillage was absent despite increased perfusion pressure, hysteroscopic fallopian tube catheterization was performed under laparoscopic visualization using the J-NCS-504070 fallopian tube recanalization set (Cook Incorporated, Bloomington, IN, USA). Following tubal cannulation, methylene blue spillage was reassessed to confirm restoration of tubal patency.

During hysteroscopy surgery under saline distension, the uterine cavity and endometrium were meticulously examined. Focal or diffuse inflammatory changes, including hyperplasia, congestion, edema, or polypoid lesions, were closely evaluated. Endometrial curettage was conducted only when such abnormalities were detected under hysteroscopy. Biopsy was not performed for patients with entirely normal hysteroscopic endometrial appearance to avoid unnecessary endometrial trauma, and the sampling decision was made by the operating surgeon based on intraoperative findings. The same hysteroscopy-guided endometrial biopsy criteria were applied to both patients with and without EMS. Immunohistochemical staining with CD138 antibody (Maxim Biotechnologies, Fuzhou, China) was subsequently carried out on collected endometrial specimens to diagnose CE. Endometrial tissue samples were embedded in paraffin, sectioned, and stained with hematoxylin and eosin (H&E) staining kit (Beijing Solarbio Science & Technology Co, Ltd., Beijing, China) according to our previous standard protocols [14]. As no universally accepted threshold exists for diagnosing CE based on CD138-positive plasma cell counts, this study defined CE as ≥5 CD138-positive plasma cells in 10 high-power fields, consistent with previously published criteria [19] and our prior study [14].

Patients with pelvic EMS complicated by proximal tubal obstruction infertility were classified based on whether intraoperative CD138 immunohistochemical examination was performed: Group A (CD138 detection group) and Group B (CD138 non-detection group). In Group A, patients diagnosed with CE via CD138 immunohistochemical staining received oral doxycycline (100 mg twice daily) for 14 days postoperatively. Doxycycline monotherapy was the first-line treatment for newly diagnosed CE at our institution, whereas combination antibiotic therapy was reserved for recurrent or persistent cases.

All patients underwent follow-up examination one month postoperatively. The decision about subsequent pregnancy plan was jointly made by the physician and patient. Given the strong desire for pregnancy among all participants and the predominance of Stage I and II EMS observed intraoperatively, patients were advised to directly attempt conception naturally within six months following surgery. For patients whose bilateral fallopian tubes remained obstructed postoperatively but who nonetheless wished to attempt to conceive naturally, their preferences were respected. Subsequent follow-up comprised regular telephone interviews. The follow-up period lasted for a minimum of 24 months or until pregnancy or pregnancy termination, with the final follow-up completed in March 2024.

### 2.3. Outcome Measures

The primary outcomes were the incidence of CE, the natural pregnancy rate, and the cumulative natural pregnancy rate. Secondary outcomes included the clinical pregnancy rate, miscarriage rate, ectopic pregnancy rate, and live birth rate. Clinical pregnancy was confirmed by transvaginal ultrasound (TVUS) of an intrauterine gestational sac with primitive cardiac activity, regardless of whether conception was achieved naturally or through assisted reproductive technology (ART). Natural pregnancy was defined as clinical pregnancy achieved through merely unprotected sexual intercourse without ART. The cumulative natural pregnancy rate referred to the proportion of women who achieved at least one natural conception during the follow-up period; once a woman conceived naturally, subsequent pregnancies were not included in the cumulative calculation. A miscarriage was defined as the loss of gestation before 28 weeks of pregnancy. Ectopic pregnancy was diagnosed via TVUS detection of a gestational sac outside the uterine cavity. Live birth was described as the delivery of a live infant after 28 weeks of gestation.

### 2.4. Statistical Analysis

All data were analyzed using SPSS version 26.0. Continuous variables were presented as medians and interquartile ranges or mean ± standard deviation (SD). Categorical variables were presented as frequency and percentage. Between-group comparisons of continuous variables were performed using the independent-samples *t*-test or the Mann–Whitney *U* test, while categorical variables were analyzed using the chi-squared test or Fisher’s exact test. Kaplan–Meier survival analysis was utilized to compare cumulative pregnancy rates between groups, with the log-rank test used to evaluate differences at various time points. Intention-to-treat (ITT) analysis was conducted by considering patients lost to follow-up. Multivariate analysis was performed using the Cox proportional hazards regression model. *p* values < 0.05 were considered statistically significant.

## 3. Results

### 3.1. Baseline Characteristics

**Figure 1:**
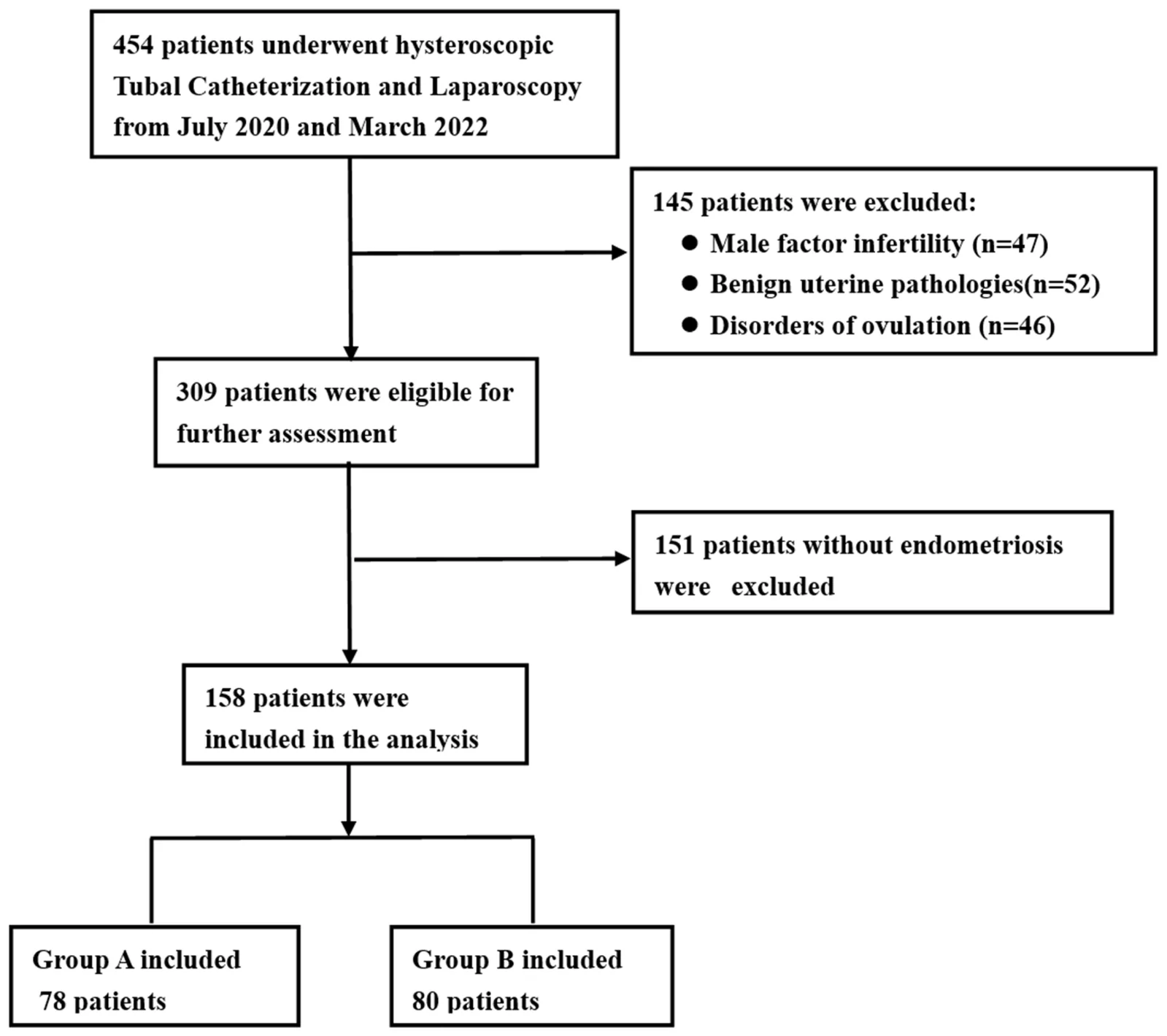
Patient inclusion flow chart.

Between July 2020 and March 2022, 454 women undergoing hysteroscopic fallopian tube catheterization combined with laparoscopy for proximal tubal obstruction-related infertility were screened for eligibility. Of these, 145 were excluded due to benign uterine pathologies, male factor infertility, or ovulatory disorders; in total, 309 patients were enrolled. Among them, 158 were diagnosed with pelvic EMS. Notably, EMS patients were further divided into Group A (*n* = 78), who underwent CD138 detection, and Group B (*n* = 80), who did not. A total of 24 participants (15.2%) were lost to follow-up (Figure 1).

The baseline characteristics are summarized in Table 1. No significant differences were observed between Group A and B in terms of age, BMI, duration of infertility, infertility type, AMH levels, fallopian tube patency, or the presence of endometrial polyps. Moreover, the proportions of EMS patients with stage I–II (83.4% vs. 88.8%) and the SUP phenotype (74.4% vs. 87.5%) were comparable between the groups.

### 3.2. Incidence of CE

**Figure 2:**
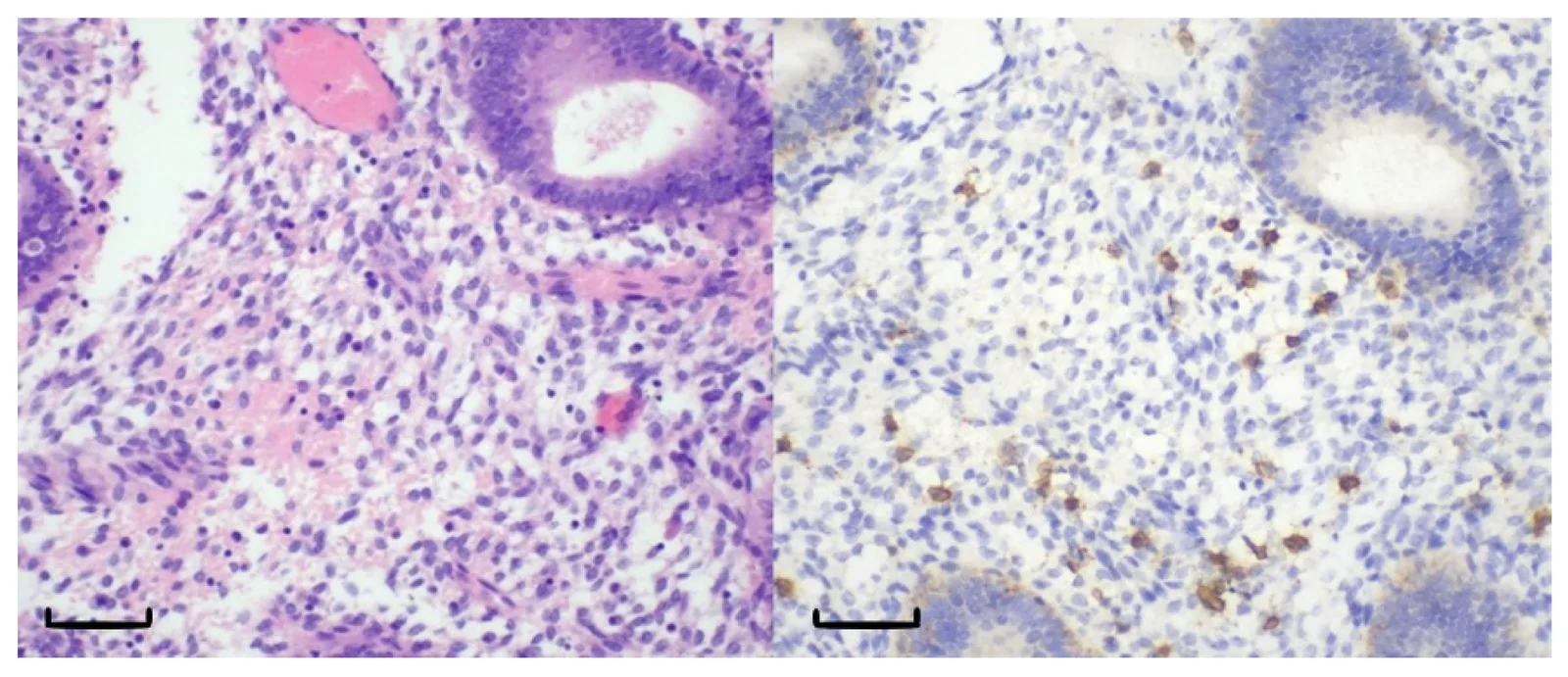
Representative Images of H&E Staining and CD138 Immunohistochemical Staining.

Within the subset of infertile patients presenting with pelvic EMS and proximal tubal obstruction, 78 cases underwent CD138 immunohistochemical staining for pathological evaluation. Among them, 49 were diagnosed with CE (Figure 2), yielding an incidence rate of 62.8% (49/78). In comparison, the incidence of CE among patients without EMS was 68.2% (58/85). However, the difference in CE incidence did not reach statistical significance (*p* > 0.05) (Appendix A Table A1).

### 3.3. Postoperative Pregnancy Outcomes

During postoperative follow-up, pregnancy outcomes were monitored, with 24 cases lost to follow-up. In accordance with the ITT principle, lost cases from either group were considered as non-pregnant outcomes. At the final follow-up, one participant in Group A was still pregnant. The clinical pregnancy rate (60.3% vs. 46.3%) and live birth rate (46.2% vs. 35.0%) were higher in Group A than in Group B, although the differences were not statistically significant. The miscarriage rate (19.1% vs. 18.9%) and ectopic pregnancy rate (1.3% vs. 2.5%) were comparable between the two groups. Notably, the natural conception rate was significantly higher in Group A compared with Group B (46.2% vs. 26.3%, *p* = 0.009) (Table 2). Among EMS patients who underwent CD138 testing, postoperative pregnancy outcomes were further stratified according to EMS women with or without CE, as shown in Supplementary Table S1. Of note, this subgroup was derived from selective hysteroscopy-guided biopsy and is prone to selection bias.

### 3.4. Postoperative Cumulative Natural Pregnancy

**Figure 3:**
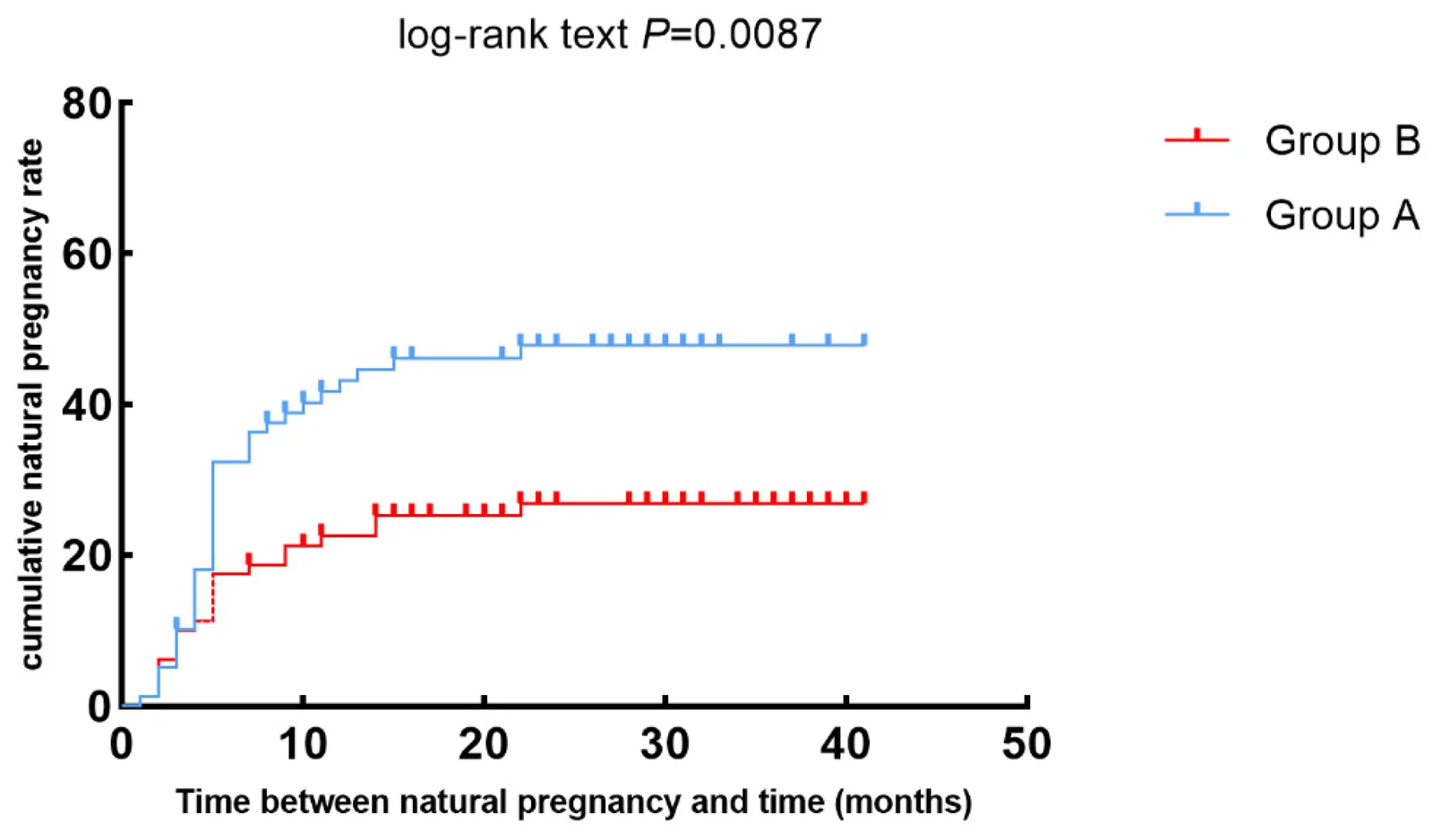
Kaplan–Meier survival analysis for cumulative natural pregnancy rate after surgery.

Following surgical intervention, patients in Group A exhibited consistently higher cumulative natural pregnancy rates at postoperative intervals of 3, 6, 12, 18, and 24 months and beyond compared to those in Group B. Notably, commencing from the 6-month postoperative mark, the disparity in cumulative natural pregnancy rates between the two groups achieved statistical significance (*p* < 0.05). (Table 3). Additionally, Kaplan–Meier survival analysis demonstrated a statistically significantly higher cumulative natural pregnancy rate in Group A compared to Group B (*p* < 0.05) (Figure 3). Importantly, multivariate Cox proportional hazards regression was performed with variables related to pregnancy, and the results showed that the diagnosis and treatment of CE was the protective factor for the cumulative natural pregnancy rate in EMS patients with proximal tubal obstruction after hysteroscopic tubal catheterization and laparoscopy (HR 1.78; 95% CI 1.06, 2.97; *p* = 0.028) (Table 4).

## 4. Discussion

Our analysis focused on infertility treatment in EMS patients with proximal tubal obstruction. The results indicated an association between CE diagnosis and antibiotic intervention and higher postoperative spontaneous pregnancy probability in patients with endometriosis-related proximal tubal obstruction.

EMS and CE are both recognized as chronic inflammatory conditions. In EMS, ectopic endometrial tissue induces a pelvic inflammatory response and local immune dysregulation, resulting in elevated levels of inflammatory cells and mediators within the pelvic fluid. These alterations contribute to pelvic adhesions and impaired tubal function by disrupting key physiological processes such as ciliary activity and smooth muscle contraction, thereby compromising tubal transport [20]. Consequently, the accumulation of secretions and tissue debris within the tubal lumen may cause functional tubal obstruction, hindering the passage of sperm or fertilized oocytes into the uterine cavity and ultimately causing infertility. Furthermore, ascending genital tract infections may induce pelvic inflammation, which can result in distal tubal obstruction and hydrosalpinx, and in severe cases, irreversible obstructive lesions of the proximal fallopian tubes [21].

Previous studies have reported that the incidence of CE is significantly higher in infertile patients with EMS [12,22,23]. In our investigation, the prevalence of CE reached 62.8% in individuals presenting with pelvic EMS and proximal tubal obstruction, which is markedly higher than that previously reported in EMS populations, ranging from 3% to 53% [24]. Such disparities may be attributed to differences in the demographic composition as well as inconsistencies in diagnostic criteria. Intrauterine inflammation has been shown to induce abnormal uterine contractility and retrograde peristalsis, thereby facilitating retrograde menstruation into the pelvic cavity and increasing susceptibility to pelvic EMS [22]. Consequently, infertile patients with concomitant proximal tubal obstruction and EMS may be particularly predisposed to CE. Moreover, previous studies have reported the coexistence of microbial infections within the uterine cavity and dysbiosis of the microbiota in both EMS and CE [11], suggesting a potential interrelationship between these conditions.

In our study, patients with proximal tubal obstruction but without EMS exhibited a higher incidence of CE compared with those with EMS (68.2% vs. 62.8%, *p* > 0.05), although the difference was not statistically significant. The discrepancy may be explained by differences in study populations. Specifically, our cohort was restricted to patients with proximal tubal obstruction, a condition that may arise from either EMS or pelvic inflammatory disease through localized inflammatory processes. Notably, tubal obstruction caused by pelvic inflammatory lesions in the absence of EMS, often due to pathogenic microbial infections, is more likely to result in tubal fibrosis and more severe structural damage to the fallopian tubes. In addition, intraoperative evaluation of endometrial tissue for inflammatory changes and subsequent diagnostic curettage for histopathological confirmation of CE are essential. However, given the lack of pathognomonic intraoperative features of CE, its diagnosis may be easily overlooked during surgery.

Recent studies have shown that infertile patients with mild EMS complicated by CE exhibit a reduced cumulative pregnancy rate postoperatively compared to those without CE (46.51% vs. 71.13%, *p* = 0.004) [14]. CE has thus been identified as an independent risk factor affecting pregnancy outcomes in infertile patients with mild EMS following laparoscopic interventions. In our study, among infertile women with pelvic EMS and proximal tubal obstruction, those identified and therapeutically managed for CE manifested a markedly elevated postoperative natural pregnancy rate compared to their unmonitored counterparts (46.2% vs. 26.3%, *p* = 0.009). Additionally, the identification and treatment of CE were protective factors for the cumulative natural pregnancy rate. Patients were advised to attempt natural conception within six months post-laparoscopy; however, some subjects continued to pursue natural conception for more than two years following surgery. Analysis of cumulative pregnancy rates revealed a marked increase within the initial six-month postoperative period for both cohorts, with patients diagnosed and treated for CE exhibiting a more pronounced rise in cumulative natural pregnancy rates beginning at the sixth postoperative month. Statistically significant differences between the two cohorts emerged thereafter, with the rate of increase slowing around nine months and eventually reaching a plateau by approximately 15 months. These findings highlight the importance of avoiding indiscriminate attempts at natural conception in this infertile population and underscore the need for timely fertility reassessment and consideration of assisted reproductive technologies when appropriate. The observed pattern may reflect the increased susceptibility to CE associated with EMS, which can contribute to infertility through fallopian tube obstruction and impaired endometrial receptivity [15]. Evidence further supports that early diagnosis and therapeutic management of CE in patients with mild EMS are associated with improved clinical pregnancy and live birth rates.

Concurrently, animal studies have demonstrated that antibiotic therapy in female murine EMS models can suppress the progression of ectopic lesions via the NF-κB signaling pathway [24], although the translational relevance to human physiology remains to be fully elucidated. Recent clinical investigations further indicate that targeted antibiotic treatment for CE can effectively reduce serum CA-125 levels in patients with ovarian EMS [25]. Collectively, these findings suggest that timely antibiotic intervention for CE in EMS patients not only alleviates the negative impact of CE on endometrial receptivity but may also contribute to limiting the initiation and progression of EMS. Thus, antimicrobial therapy may serve a dual function, simultaneously addressing CE while exerting inhibitory effects on EMS progression.

Our study was a single-center retrospective analysis, with histopathological examinations performed by specialized pathologists using CD138 immunohistochemistry, ensuring high diagnostic consistency. However, our study had several limitations inherent to its single-center retrospective design. First, CD138 staining was performed only in a subset of EMS patients, and post-treatment biopsy was not routinely performed to confirm CE resolution. Thus, the true prevalence of CE in the control group remains unknown, potentially introducing selection bias and confounding the association between CE management and spontaneous pregnancy outcomes. These findings should be interpreted cautiously. Second, there remains no universally agreed histological threshold for CE diagnosis using CD138 staining. Histopathological assessment relied on intraoperative hysteroscopic appearance, which lacks sensitivity for CE diagnosis. Thirdly, even after multivariate adjustment, residual confounding from baseline imbalances cannot be fully ruled out. Future prospective studies with standardized uniform endometrial sampling for all subjects are warranted to confirm our findings.

In summary, diagnosis and antibiotic-directed treatment of CE were associated with higher postoperative spontaneous pregnancy rates in infertile women with EMS and proximal tubal obstruction, underscoring the potential value of CE management in this population. Given the retrospective design and potential for selection bias and residual confounding, these findings should be interpreted cautiously.

## Figures and Tables

**Table 1:** Baseline clinical characteristics of participants.

Characteristic	Group A (*n* = 78)	Group B (*n* = 80)	*p*-Value
Patient age (years)	29.85 ± 2.91	30.09 ± 3.03	0.610
BMI (kg/m^2^)	21.26 ± 2.70	21.24 ± 2.56	0.956
Duration of infertility *n* (%)			
≤3 years	52 (66.7)	52 (65.0)	0.825
>3 years	26 (33.3)	28 (35.0)	
Infertility type *n* (%)			
Primary infertility	38 (48.7)	33 (41.3)	0.345
Secondary infertility	40 (51.3)	47 (58.8)	
AMH (ng/mL)	4.42 ± 2.78	4.53 ± 2.61	0.798
r-AFS total score	7.99 ± 12.14	6.84 ± 11.52	
r-AFS staging			0.709
Stage I	57 (73.1)	62 (77.5)	
Stage II	8 (10.3)	9 (11.3)	
Stage III	9 (11.5)	5 (6.3)	
Stage IV	4 (5.1)	4 (5.0)	
Endometriosis phenotypes *n* (%)			0.102
SUP	58 (74.4)	70 (87.5)	
OMA	17 (21.8)	9 (11.3)	
DIE	3 (3.8)	1 (1.3)	
Patency of fallopian tubes after surgery *n* (%)			0.605
Bilateral	59 (75.6)	62 (77.5)	
Unilateral	17 (21.8)	14 (17.5)	
Neither	2 (2.6)	4 (5.0)	
Presence of uterine endometrial polyps *n* (%)	32 (41.0)	25 (31.3)	0.201

BMI, Body Mass Index; AMH, Anti-Müllerian hormone; r-AFS, Revised American Fertility Society; SUP, superficial peritoneal endometriosis; OMA, ovarian endometriosis; DIE, deeply infiltrating endometriosis.

**Table 2:** Postoperative pregnancy outcomes between groups.

Pregnancy Outcomes	Group A (*n* = 78)	Group B (*n* = 80)	*p*-Value
Clinical pregnancy rate	60.3% (47/78)	46.3% (37/80)	0.078
Natural pregnancy rate	46.2% (36/78)	26.3% (21/80)	0.009 *
Miscarriage rate	19.1% (9/47)	18.9% (7/37)	0.979
Ectopic pregnancy rate	1.3% (1/78)	2.5% (2/80)	0.422
Live birth rate	46.2% (36/78)	35.0% (28/80)	0.153

* *p* < 0.05.

**Table 3:** Postoperative cumulative natural pregnancy rates between groups.

Postoperative Time	Group A (*n* = 78)	Group B (*n* = 80)	*p*-Value
3 months	10.3% (8/78)	10.0% (8/80)	0.967
6 months	32.1% (25/78)	17.5% (14/80)	0.042 *
12 months	42.3% (33/78)	22.5% (18/80)	0.009 *
18 months	44.9% (35/78)	25.0% (20/80)	0.009 *
24 months and beyond	46.2% (36/78)	26.3% (21/80)	0.009 *

* *p* < 0.05.

**Table 4:** Multivariate Cox regression of variables related to cumulative natural pregnancy rates.

Variables	HR	95% CI	*p*-Value
Diagnosis and treatment of CE	1.78	1.06–2.97	0.028 *
Age	0.98	0.89–1.07	0.731
BMI (kg/m^2^)	0.87	0.78–0.97	0.013
Duration of infertility	0.78	0.44–1.39	0.409
Infertility type	1.22	0.72–2.07	0.461
r-AFS staging	0.87	0.35–2.14	0.754
Endometriosis phenotypes	1.53	0.55–4.25	0.413
Patency of fallopian tubes	0.97	0.86–1.05	0.907
Endometrial polyps	1.10	0.64–1.86	0.738

CE, chronic endometritis; BMI, Body Mass Index; r-AFS, Revised American Fertility Society; * *p* < 0.05.

## Data Availability

The data supporting the findings of this study are included in the article and its Supplementary Materials. Further inquiries may be directed to the corresponding authors and data will be made available upon reasonable request.

## Supplementary Materials

The following supporting information can be downloaded at: https://www.mdpi.com/article/10.3390/jcm15176845/s1, Table S1: Postoperative pregnancy outcomes stratified by chronic endometritis status among endometriosis patients under CD138 staining.

## Appendix A

**Table A1:** Baseline clinical characteristics of participants who underwent CE138 immunohistochemical staining after hysteroscopic tubal catheterization and laparoscopy.

Characteristic	Endometriosis (*n* = 78)	Without Endometriosis (*n* = 85)	*p*-Value
Patient age (years)	29.85 ± 2.91	29.61 ± 4.01	0.920
BMI (kg/m^2^)	21.26 ± 2.70	22.10 ± 3.20	0.127
Duration of infertility *n* (%)			
≤3 years	52 (66.7)	58 (68.2)	0.831
>3 years	26 (33.3)	27 (31.8)	
Infertility type *n* (%)			
Primary infertility	38 (48.7)	37 (43.5)	0.507
Secondary infertility	40 (51.3)	48 (56.5)	
AMH (ng/mL)	4.42 ± 2.78	4.56 ± 3.52	0.393
Patency of fallopian tubes after surgery *n* (%)			
Bilateral	59 (75.6)	45 (52.9)	0.001 *
Unilateral	17 (21.8)	22 (25.9)	
Neither	2 (2.6)	18 (21.2)	
Presence of uterine endometrial polyps *n* (%)	32 (41.0)	16 (18.8)	0.002 *
Diagnosis with CE	49 (62.8)	58 (68.2)	0.467

BMI, Body Mass Index; AMH, Anti-Müllerian hormone; CE, chronic endometritis; * *p* < 0.05.
