# Supplementary material for: Diagnosis and Management of Chronic Endometritis: Effects on Postoperative Pregnancy Outcomes in Endometriosis Patients with Proximal Tubal Obstruction Undergoing Hysteroscopic Tubal Catheterization and Laparoscopy
Source: J Clin Med. 2026 Sep 3;15(17):6845. doi: 10.3390/jcm15176845 (PMC13566601; doi:10.3390/jcm15176845)
Supplement: Supplementary file 1 [file jcm-15-06845-s001.zip › jcm-4505478-supplementary.pdf]

Table S1. Postoperative pregnancy outcomes stratified by chronic endometritis status among endometriosis patients under CD138 staining.

**Pregnancy Outcomes**	**EMS patients with CE (*n*=49)**	**EMS patients without CE (n=29)**	***p*-Value**
clinical pregnancy rate	59.2% (29/49)	62.1% (18/29)	0.801
natural pregnancy rate	44.9% (22/49)	48.3% (14/29)	0.772
Miscarriage rate	24.1% (7/29)	11.1% (2/18)	0.324
ectopic pregnancy rate	2.0% (1/49)	0% (0/29)	0.439
live birth rate	42.9% (21/49)	51.7% (15/29)	0.448

CE, chronic endometritis; EMS, endometriosis.
